# Embryonal Control of Yellow Seed Coat Locus *ECY1* Is Related to Alanine and Phenylalanine Metabolism in the Seed Embryo of *Brassica napus*

**DOI:** 10.1534/g3.116.027110

**Published:** 2016-02-18

**Authors:** Fulin Wang, Jiewang He, Jianghua Shi, Tao Zheng, Fei Xu, Guanting Wu, Renhu Liu, Shengyi Liu

**Affiliations:** * State Key Laboratory Breeding Base for Zhejiang Sustainable Pest and Disease Control, Zhejiang Provincial Key Laboratory of Genetic Engineering on Plant Metabolism, Institute of Virology and Biotechnology, Zhejiang Academy of Agricultural Sciences, Hangzhou 310021, China,; † Research and Development Center, Hubei Tobacco China Industrial Company, Wuhan 430040, China; ‡ Institute of Digital Agriculture, Zhejiang Academy of Agricultural Sciences, Hangzhou 310021, China,; § Key Laboratory of Biology and Genetic Improvement of Oil Crops, the Ministry of Agriculture, Oil Crops Research Institute of the Chinese Academy of Agricultural Sciences, Wuhan 430062, China

**Keywords:** *Brassica napus*, yellow seed coat, xenia effect, free amino acids, transcriptome sequencing

## Abstract

Seed coat color is determined by the type of pigment deposited in the seed coat cells. It is related to important agronomic traits of seeds such as seed dormancy, longevity, oil content, protein content and fiber content. In *Brassica napus*, inheritance of seed coat color is related to maternal effects and pollen effects (xenia effects). In this research we isolated a mutation of yellow seeded *B. napus* controlled by a single Mendelian locus, which is named *Embryonal Control of Yellow seed coat 1* (*Ecy1*). Microscopy of transverse sections of the mature seed show that pigment is deposited only in the outer layer of the seed coat. Using Illumina Hisequation 2000 sequencing technology, a total of 12 GB clean data, 116× coverage of coding sequences of *B. napus*, was achieved from seeds 26 d after pollination (DAP). It was assembled into 172,238 independent transcripts, and 55,637 unigenes. A total of 139 orthologous genes of Arabidopsis *transparent testa* (*TT*) genes were mapped *in silico* to 19 chromosomes of *B. napus*. Only 49 of the *TT* orthologous genes are transcribed in seeds. However transcription of all orthologs was independent of embryonal control of seed coat color. Only 55 genes were found to be differentially expressed between brown seeds and the yellow mutant. Of these 55, 50 were upregulated and five were downregulated in yellow seeds as compared to their brown counterparts. By KEGG classification, 14 metabolic pathways were significantly enriched. Of these, five pathways: phenylpropanoid biosynthesis, cyanoamino acid metabolism, plant hormone signal transduction, metabolic pathways, and biosynthesis of secondary metabolites, were related with seed coat pigmentation. Free amino acid quantification showed that Ala and Phe were present at higher levels in the embryos of yellow seeds as compared to those of brown seeds. This increase was not observed in the seed coat. Moreover, the excess amount of free Ala was exactly twice that of Phe in the embryo. The pigment substrate chalcone is synthesized from two molecules of Ala and one molecule of Phe. The correlation between accumulation of Ala and Phe, and disappearance of pigment in the yellow seeded mutant, suggests that embryonal control of seed coat color is related with Phe and Ala metabolism in the embryo of *B. napus*.

In *Brassica* species, seed coat color can be black, yellow, brown, or mosaic (Yu 2013). Seed coat color is of considerable interest as it is associated with oil content, meal quality, and oil brightness. Yellow seeds have a significantly thinner seed coat than black seeds; consequentially, they have a lower hull proportion, and a higher proportion of oil and protein (Mahmood *et al.* 2006). Light seed color and low fiber content coincide as the biochemical pathways leading to lignin and pigment synthesis have common precursors (Whetten *et al.* 1998). Some QTL controlling seed coat color and acid detergent fiber content were also found to colocate in *Brassica napus* (Badani *et al.* 2006; Liu *et al.* 2012). The higher protein and lower fiber content in yellow oilseeds improves the meal quality for poultry and livestock. Additionally, the yellow seeds produce more transparent oil, which has a higher market value. Thus, the development of cultivars with yellow seed coat is an important goal for the breeding of *Brassica* oil crops.

The inheritance of seed coat color in *B. napus* is reported to be related to maternal and embryonal control, which determine seed coat color by the maternal and the embryonal genotype, respectively (Heneen and Brismar 2001). With maternal control, a single plant produces seeds all of the same color. With embryonal control, a single plant may produce a mixture of seeds with different colors. In the most reported cases, the inheritance of seed coat color in *B. napus* exhibits maternal control. Three or four independent recessive genes have been mapped for yellow seed color determination in *B. napus* (Rahman *et al.* 2001, 2010). A partially dominant gene has also been identified for control of yellow seed color in crosses between black-seeded and resynthesized yellow-seeded *B. napus* (Liu *et al.* 2005; Xiao *et al.* 2007). From an F_2_ population derived from Youyan 2 × GH, two independent QTL, with dominant and recessive effects, respectively, were discovered for yellow seed color inheritance (Liu *et al.* 2006). Recently, the dominant QTL was fine-mapped on chromosome A9, and the candidate gene was identified as *Cinnamoyl Co-A Reductase 1* (*CCR1*) (Liu *et al.* 2012). All of these well characterized loci are maternal controlling loci.

In the model plant *Arabidopsis*, 27 genes involved in the flavonoid (flavonols and proanthocyanidin) biosynthetic pathway have been elucidated using different *transparent testa* (*tt*) mutations (Yu 2013; Albert *et al.* 2014). All of these genes exert maternal controlling effects as well. These genes establish a reference model to help understand the determination of yellow seed coat color in *B. napus*. However, the genomes of *Brassica* species are much more complex than that of *Arabidopsis*. Homologs of these genes in *Brassica* species are often multiple (Lysak *et al.* 2005; Chai *et al.* 2009). Although some single gene mutations of these homologs result in yellow seed coat in some genetic backgrounds (Zhang *et al.* 2009), successful modification of seed coat color by genetic manipulation of these *tt* homologs in *B. napus* has not been widely reported. In particular, the embryonal control of seed coat color is still not well characterized in *B. napus*.

To date, most of the genes or alleles from yellow seeded parents are recessive, and have shown maternal inheritance. Although dominant alleles (Xiao *et al.* 2007; Liu *et al.* 2012) and embryonal inheritance (Heneen and Brismar 2001) have also been reported, the genetic mechanism determining the embryonal control of yellow seeds is still unknown. In this study, we isolated a dominant mutation of yellow seeded *B. napus* controlled by a single Mendelian locus with embryonal effect. Alanine and phenylalanine metabolism in the seed embryo of *B. napus* were found to be related to the embryonal control of seed coat color.

## Materials and Methods

### Plant materials

**Figure 1:**
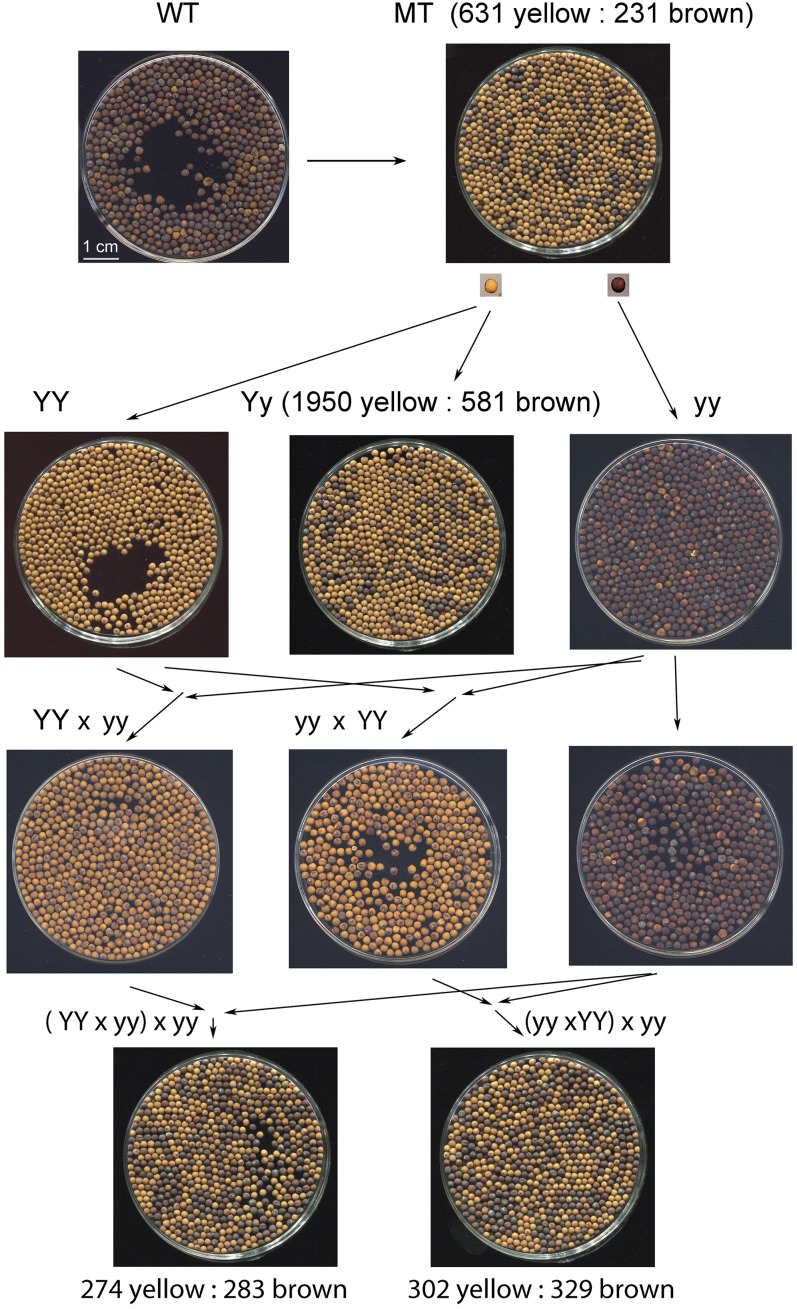
Inheritance of the yellow-seeded mutant of *Brassica napus*.

The *Brassica napus* brown-seeded inbred line Cy2B (bagged for more than 10 generations), and its yellow-seeded near-isogenic line Cy2Y, were used in this study. The plants were grown in the field in Hangzhou, China, under normal cultivation condition. Crosses were made by manual pollination. Inflorescences on the first lateral branches were selected for crosses. Opened flowers and small buds were removed. Hands and forceps were sponged with a cotton ball soaked in 75% alcohol, then stamens in large flower buds, estimated to be 1–2 d before anthesis, were removed using the cleaned forceps. The pollen from the bagged paternal plants was hand-pollinated onto the emasculated pistils. The hand-pollinated inflorescences were then labeled and covered in small paper bags for 5–7 d for seed production, and then uncovered until maturity. Seed coat color was distinct and easy to judge by eye (Figure 1).

### Microscopy of seed coat section

Seeds of *B. napus* were embedded in tissue freezing medium (Jung, Leica), frozen at –20° overnight, and then cut into 20 μm slices using a freezing microtome (Leica CM1950). The sections were then loaded onto glass slides for examination by microscope (Nikon Eclipse NI-U). For transmission electron microscopy, fresh mature seeds isolated from plants in the harvest season, were cut into small pieces, fixed in 2.5% (w/v) glutaraldehyde in phosphate buffer (0.1 M, pH 7.2) overnight at 4°, rinsed, and incubated in 1% (w/v) OsO4 in 0.1 M sodium phosphate buffer (pH 7.2) for 24 h at 4°. The samples were then rinsed again in phosphate buffer (0.1 M, pH 7.2), dehydrated in ethanol series, infiltrated with a graded series of Spurr’s resin in acetone, and then embedded in Spurr’s resin. The sections were obtained using a diamond knife with a Reichert OM2 ultramicrotome, and then stained in 2% (w/v) uranyl acetate (pH 5) followed by 10 mM lead citrate (pH 12), and viewed with a transmission electron microscope (JEM-1230, JEOL) (Shi *et al.* 2015).

### Seed transcriptome sequencing and assembly

The near isogenic lines Cy2B and Cy2Y were prepared from the single heterozygous plant (*Yy* genotype) self crossed for five consecutive years. Total RNA was extracted from the field-grown seeds 26 DAP of Cy2Y and Cy2B, respectively, using TRIzol Regent (Life Technologies) following the manufacturer’s instructions. Quality of total RNA was checked by agarose gel electrophoresis and Agilent Bioanalyzer 2100 (Agilent Technologies). Qualified total RNA was purified using an RNeasy micro kit (Qiagen, Germany), and an RNase-Free DNase set (Qiagen). Poly (A) mRNA was then isolated using an Oligotex mRNA mini kit (Qiagen). Two pair-end cDNA libraries from brown seeds (Cy2B), and yellow mutation seeds (Cy2Y), respectively, were constructed using the Genomic Sample Prep kit (Illumina). Short fragments were purified with the Qubit dsDNA HS Assay kit (Invitrogen). Each of the two libraries had an average insert size of 400 bp, and 100 bp from each end of the insert was sequenced using Illumina HiSeq 2000. All the RNA-seq raw data in this study has been submitted to the NCBI Sequence Read Archive (SRA), under the accession number SRA082456. All the cDNA library construction and sequencing services were provided by Novogene (Beijing, China).

Raw reads from the *de novo* sequencing were filtered into clean reads by removing reads containing adapter, reads containing more than 10% N, or containing more than 50% low quality bases (sQ ≤ 5). The clean reads were assembled using the software Trinity version V2012-10-05, (Grabherr *et al.* 2011), with the parameter min_kmer-cov set to 2. All other parameters were default.

Gene expression was quantified by normalizing the number of raw reads mapped to the assembled unigenes. The transcription level of each gene was calculated as RPKM (Reads Per Kilobase per Million mapped Reads) values (Mortazavi *et al.* 2008). The significance of difference in gene expression between brown and yellow mutants was determined using DEGseq, an R package (Wang *et al.* 2010). False discovery rate (FDR) was applied to identify the threshold of P value in multiple tests (Buschmann *et al.* 2014). When FDR is less than 0.05, and Log_2_ ratio is greater than 1 (twofold change), the unigenes were considered as differentially expressed. The analysis service was provided by Novogene.

### Functional annotation

Function of the assembled genes was annotated according to the databases of NR, NT, Swiss-Prot, Pfam, and KEGG as previously described (Liu *et al.* 2013). NCBI blast was used for alignment against NR, NT, and Swiss-Prot with E-value thresholds of 10^−5^. The HMMER 3.0 package was used for Pfam database scanning. KEGG pathways were assigned to the unigenes by the single-directional best hit (SBH) method, on the KEGG automatic annotation server (KAAS) online (Moriya *et al.* 2007). The analysis service was provided by Novogene.

### Amino acid quantification

Free amino acid quantification was performed as described in our previous paper (Shi *et al.* 2015). The embryo and testa of 32 DAP seeds were dissected on ice. About 300 μg fresh tissue was immediately mixed with 1 ml 8% (w/v) 5-sulfosalicylic acid in a 2 ml Eppendorf tube, then homogenized in tissue lyser Retsch MM400 (Retsch, Germany). After 1 h of extraction at 4°, the homogenized samples were centrifuged for 10 min at 12,000 *g*. Then supernatants were filtered through 0.22 μm filters. Free amino acids were measured with an automated amino acid analyzer (Hitachi L-8900).

### Quantitative RT-PCR (qRT-PCR) analysis

The primers were designed using the software Primer3Plus. Total RNA was extracted from 100 mg fresh seeds, 26 DAP, using 1 ml TRIzol. About 1 μg of total RNA was treated with RNase free DNase (Promega) before reverse transcription. qRT-PCR was performed as described previously (Liu *et al.* 2010). *BnAct2* (*BnaA10g22340D*) and *BnUbq5* (*BnaA07g19250D*) genes were used as internal controls for normalization. Supplemental Material Table S4 lists the primers used in this study.

### Data availability

File S1 contains Supplementary Table S1 to S3, Supplementary Figure S1 to S3 and detailed descriptions of all supplemental files. File S2 contains Supplementary Table S4, gene specific primers used for qPCR. File S3 contains Supplementary Table S5, free amino acids content in seeds. File S4 lists all the *Arabidopsis tt* genes and the orthologs in *B*. *napus*. RNA-seq data are available at SAR with the accession number: SRA082456.

## Results

### Isolation of a yellow-seeded mutant of B. napus controlled by embryo genotype

In 2006, we found a mutant of yellow-seeded plants of *B. napus* in the field. The mutant plant produced distinct yellow seeds, as compared to the brown seeds of the wild type (Figure 1). The bagged seeds of the mutant plant were composed of a mixture of brown and yellow seeds (Figure 1) in the proportions of 631 yellow seeds to 231 brown seeds. The ratio fits well with the monogenic segregation ratio 3:1 (*χ*^2^ = 1.48, *P* = 0.22), suggesting that the yellow mutation is controlled by a single Mendelian element. Commonly, three types of segregation found in seed: embryo, gametophyte, and maternal. Maternal control of seeds segregation would cause single plants to produce nonsegregated seeds. Gametophyte segregation would cause 1:1 segregation of seeds (*χ*^2^ = 185.6, *P* < 0.001). So this segregation data eliminated the choices of maternal and gametophyte segregation type and accepted the embryo segregation type.

In the autumn of 2007, yellow and brown seeds obtained from the mutant plant were isolated and sown in different field blocks. In the brown seed block, all the brown seeds gave rise to pure brown-seeded plants. In the yellow seed block, 13 yellow seeds gave rise to pure yellow-seeded plants, but the other 21 yellow seeds gave rise to plants that yielded a mixture of yellow and brown seeds. The ratio of 21 heterozygous yellow seeds *vs.* 13 homozygous yellow seeds agreed with the expected ratio of 2:1 (*χ*^2^ = 0.37, *P* = 0.54) with the monogenic model. The progeny from one heterozygous yellow seed-derived plant comprised 1950 yellow seeds and 581 brown seeds (Figure 1), in a ratio of 3:1 (*χ*^2^ = 0.056, *P* = 0.81), which corresponds to the expected ration for a monogenic trait. The results supported the hypothesis that the trait of yellow seeds is heritable, and controlled by a single dominant locus. We harvested the unsegregated pure yellow lines and pure brown lines, and gave the pure line names Cy2Y (genotype: *YY*) and Cy2B (genotype: *yy*), respectively. Because Cy2Y was mutated from Cy2B without substantial mutation induction, they were considered to be a near isogenic line pair.

In 2008, direct and reciprocal crosses were made between Cy2Y and Cy2B, respectively. Both the F_1_ seeds of direct (Cy2Y × Cy2B; *YY* × *yy*) and reciprocal (Cy2B × Cy2Y; *yy* × *YY*) crosses, harvested from the maternal parent (ahead of the cross symbol), were composed of pure yellow seeds (Figure 1). The results confirmed that the yellow allele was dominant over the brown allele, without maternal effect, in this genetic background. In the spring of 2009, the F_1_ plants were backcrossed to the parents of Cy2B and Cy2Y, respectively. In the backcross (Cy2Y × Cy2B) × Cy2B, the seeds of BC_1_F_1_ showed segregated seed color (Figure 1). One portion of the BC_1_F_1_ seeds was composed of 274 yellow and 283 brown seeds (*H*_0_ = 1:1, *χ*^2^ = 0.70, *P* = 0.40). Similar results were achieved from the BC_1_F_1_ of (Cy2B × Cy2Y) × Cy2B (Figure 1). The segregation of seeds from a single plant indicated that the seed coat color was controlled by the embryo genotype rather than by the maternal plant. The backcrosses also confirmed that the yellow-seeded mutation was under the control of a single locus whose function was controlled by the embryo. Hereafter, we name the yellow mutation *Embryonal Control of Yellow seed coat 1* (*Ecy1*).

### Pigment is deposited in the outer layer of the seed coat in B. napus

**Figure 2:**
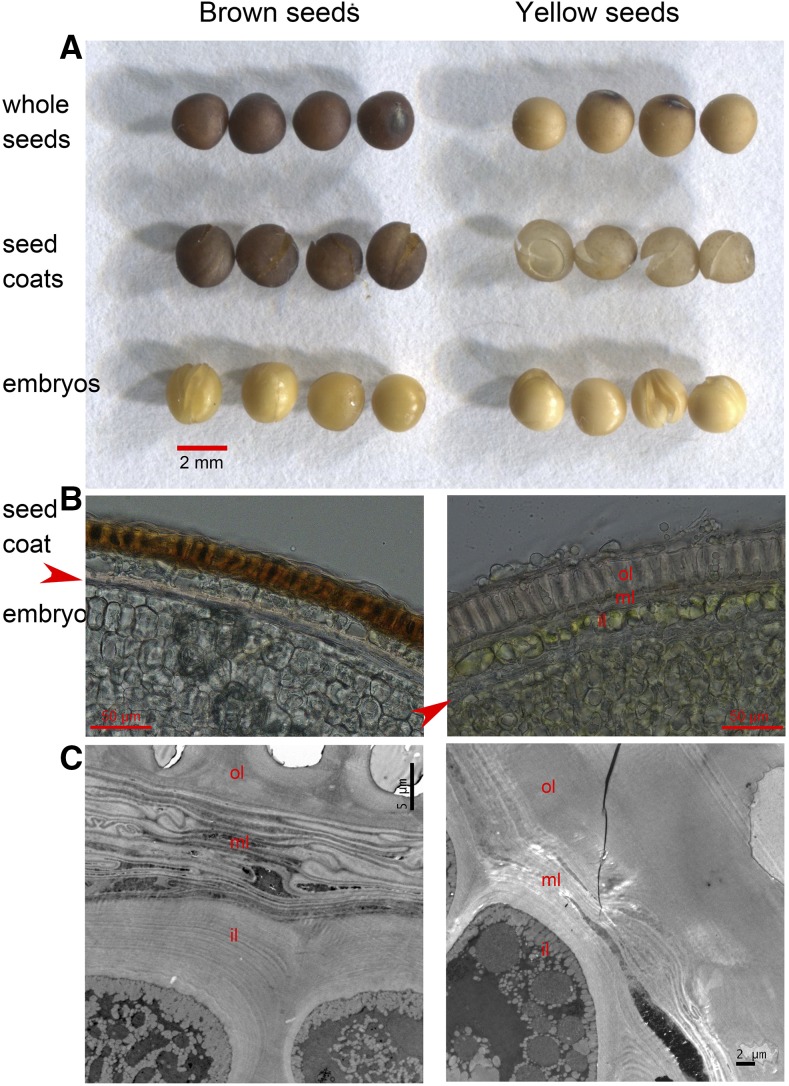
Dissected seed coat and embryo of brown and yellow seeds (A), and optical (B) and transmission electron (C) microscopy of transverse sections of the seed coats from brown and yellow seeds. Red arrows indicate the gap between seed coat and embryo. Seed coat is indicated with outer layer (ol), middle layer (ml), and inner layer (il).

Dissecting seed coat and embryo of brown and yellow seeds shows that the seed color is mainly determined by the seed coat color, not by the embryo color. Seed coat of brown seeds and yellow seeds is dark brown and light yellow, respectively. Seed embryos of both brown seeds and yellow seeds are yellow, except the color is slightly lighter on yellow seed embryos than on the brown seed embryos (Figure 2A). Microscopy of transverse sections of the seeds shows that the seed coat structure of *B. napus* is quite different to the seed testa of Arabidopsis (Figure 2B). The structure of the Arabidopsis seed testa is composed of an epidermis layer, a palisade layer, a crushed parenchymatic layer, an endothelium layer, and an aleurone layer (Debeaujon *et al.* 2000). The seed coat of *B. napus* is composed of three cell layers: an outer layer with cells staking regularly (Figure 2B), a middle layer with crushed parenchymatous cells (Figure 2C), and an inner layer with thickened cell wall (Figure 2C). Under the seed coat lie the embryo cells. The pigments responsible for the different color of seed coat in *B. napus* are deposited in the outer layer of the seed coat (Figure 2B). The structure is different to that of Arabidopsis seeds, in which proanthocyanidins are localized in the endothelium layer and the crushed parenchyatic layers, which are the inner cell layers of the seed coat (Debeaujon *et al.* 2000). The brown pigment deposited cell layer was also different in black seeds of *B. napus* (Deng *et al.* 2012).

### Seed transcriptome sequencing, de novo assembly, and annotation

A total of 31,060,834 and 30,823,125 paired-end clean reads of 100 bp in length each were generated from Cy2B and Cy2Y, respectively (Table S1). According to the genome sequence of *B. napus* (Chalhoub *et al.* 2014), 101,040 genes were annotated, with a total length of 106.6 Mb of coding sequences. Using Trinity software, the 12.38 GB clean raw bases, *i.e.*, 116× coverage of coding sequences of *B. napus*, were assembled into 172,238 transcripts and 55,637 unigenes (Table S2). The assembled transcripts varied from 201 bp to 16,406 bp, with a mean of 1155 bp, a N50 of 1699 bp, and a N90 of 558 bp. The unigenes varied from 201 bp to 16,406 bp as well, with a mean of 784 bp, a N50 of 1414 bp, and a N90 of 292 bp. Using all the assembled transcripts as a reference, 77.72% and 77.18% of clean reads from Cy2B and Cy2Y, respectively, were mapped to the reference.

Of the 172,238 assembled transcripts, 69.90% had hits in the annotated genes of *B. napus* cv. Darmor-*bzh* with Megablast thresholds e-value <1e–50 and identity > 95%. Of the 55,637 unigenes, 74.57% had annotation information in one of the following databases: NR, NT, KO, SwissProt, Pfam, GO, and KOG (Table S3). Functional classification of the genes showed that the most frequently annotated genes in seeds were classified into functional categories of cellular process (60.3%), binding (57.5%), and metabolic process (56.9%) by GO classification (Figure S1); general functional prediction (16.1%), post-translational modification (11.7%), and signal transduction (9.0%) by COG classification (Figure S2); and carbohydrate metabolism (9.4%), signal transduction (8.6%), and translation (8.6%) by KEGG classification (Figure S3). The functional annotation profile in seeds of *B. napus* is similar to those in seed coat of *B. juncea* (Liu *et al.* 2013).

### Transcription level of the genes in brown seeds and yellow mutation

**Figure 3:**
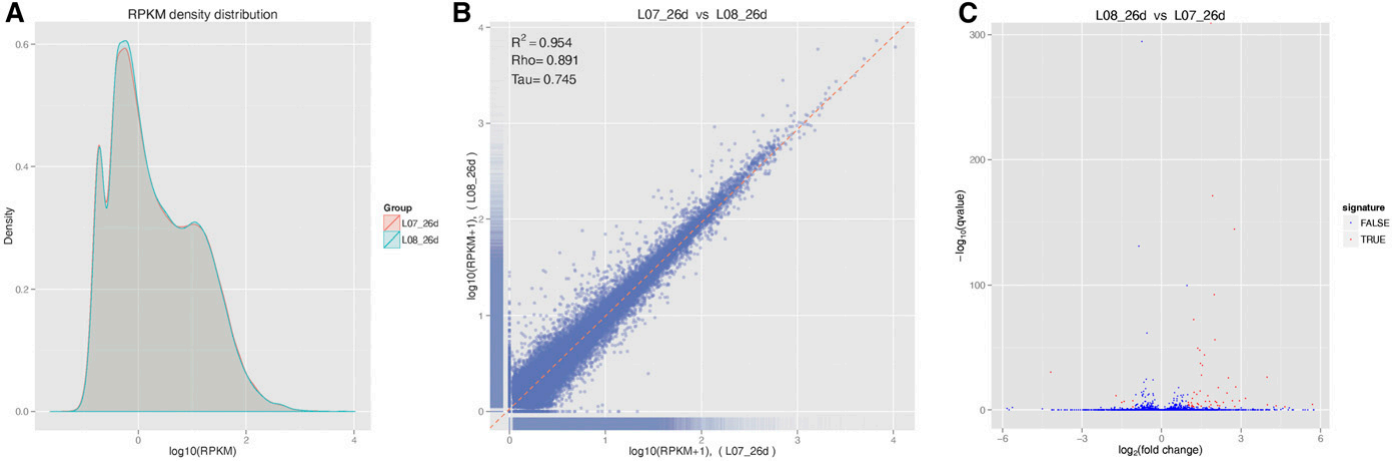
Comparison of the transcription levels between yellow (L08_26d) and brown (L07_26d) seeds transcriptome. (A) Distribution of the transcription levels. (B) Correlation of the transcription levels. (C) Significantly regulated genes (red dotted).

**Figure 4:**
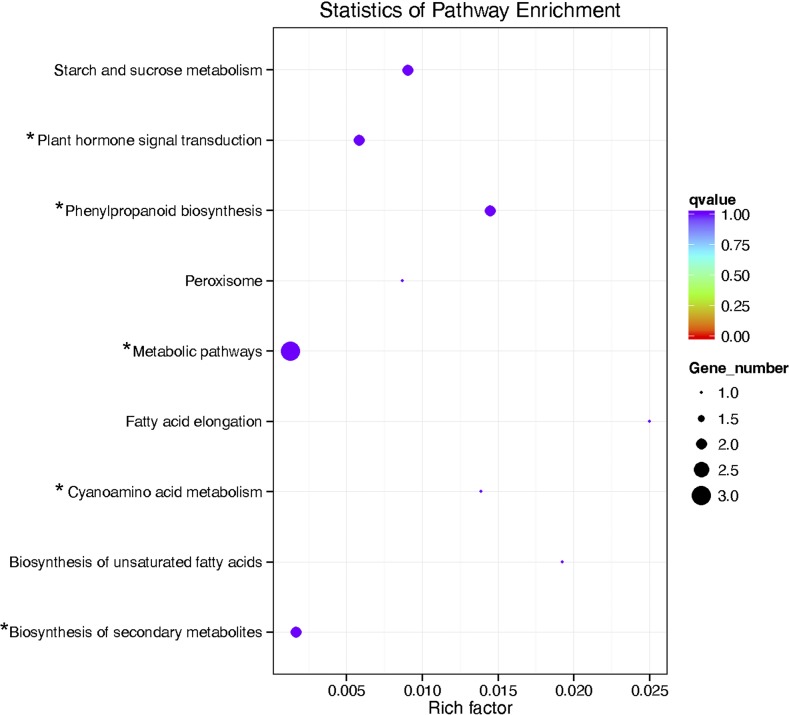
KEGG pathway enrichment of the differentially expressed genes. Asterisk (*) indicates the known pathways for pigment metabolism.

Of the 55,637 unigenes identified in the seed, transcription levels, calculated as RPKM, varied from zero to 10,484.2, with a median of 2.17 (Figure 3A). The three most abundantly transcribed genes in the seeds encode cysteine proteinase, seed specific protein Bn15D12A, and pre-mRNA-splicing factor. The distribution and correlation between transcription levels in yellow and brown seeds are shown in Figure 3. The results show that the transcription profiles of the yellow seeds and the brown were highly similar, indicating that brown and yellow seeds are highly genetically identical. Only 55 genes (Figure 3C) were found to be differentially expressed between the materials, of which 50 were upregulated (Table S4), and 5 were downregulated in the yellow seeds as compared to their brown counterparts. Relative expression of the upregulated genes varied from 2.02- to 52.07-fold, while relative expression of the downregulated genes varied from 0.30- to 0.47-fold. Compared to the 502 upregulated and 1304 downregulated genes in the seed coat of yellow and brown *B. juncea* (Liu *et al.* 2013), the number of differentially expressed genes was quite small. The most enriched pathway terms are shown in Figure 4. Five of the 14 pathways were directly related to seed coat pigmentation. Cyanoamino acid metabolism and phenylpropanoid biosynthesis pathways were especially prominent.

### Free amino acids are associated with the embryonal control of seed coat color inheritance

**Figure 5:**
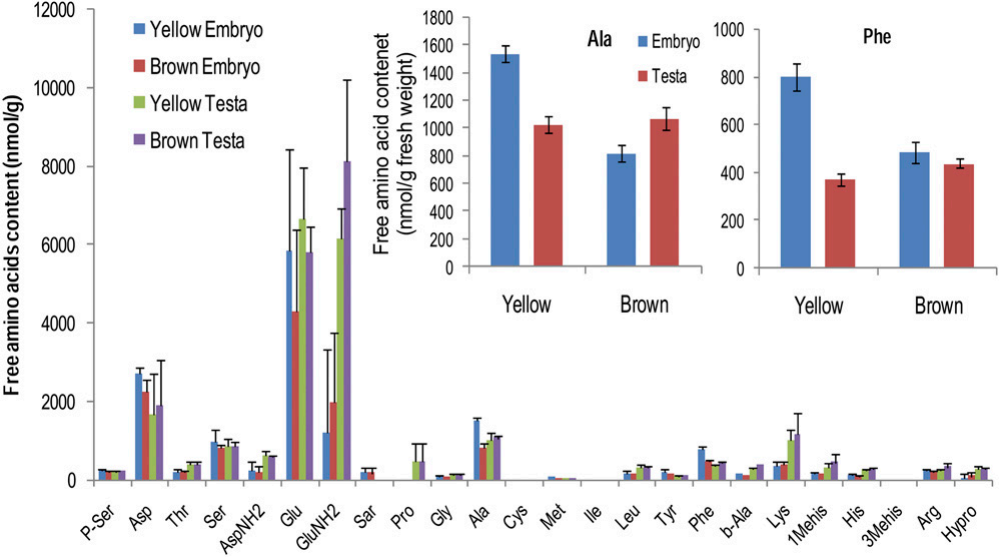
Free amino acids content in embryo and testa, respectively, in yellow and brown seeds (32 DAP).

Free amino acids are the initial substrates for pigment metabolism. In order to reveal the connection between free amino acids metabolism and seed coat color, we investigated free amino acids content, in both the embryo and the testa (32 DAP), of yellow seeds and brown seeds. A total of 40 free amino acid related fractions was analyzed (Table S5). Of these, 24 major amino acids are shown in Figure 5 for comparison. Two amino acids, alanine (Ala) and phenylalanine (Phe), showed significantly different levels in the embryo, but similar levels in the testa between yellow and brown seeds. In the embryo of yellow and brown seeds, Ala content was 1537.7 ± 59.3 and 821.8 ± 122.1 nmol/g fresh tissue, respectively. While in the testa, Ala was 1029.4 ± 178.3 nmol/g in yellow, and 1071.5 ± 79.0 nmol/g in brown. For Phe, the content in yellow embryo, yellow testa, brown embryo, and brown testa was 801.2 ± 57.1, 486.1 ± 45.2, 371.0 ± 11.3, and 439.6 ± 19.2 nmol/g, respectively. The results indicate that the free amino acids Ala and Phe are associated with the embryonal control of seed coat color in *B. napus*.

### Transcription of transparent testa gene orthologs is independent of the embryonal control of seed coat color

**Figure 6:**
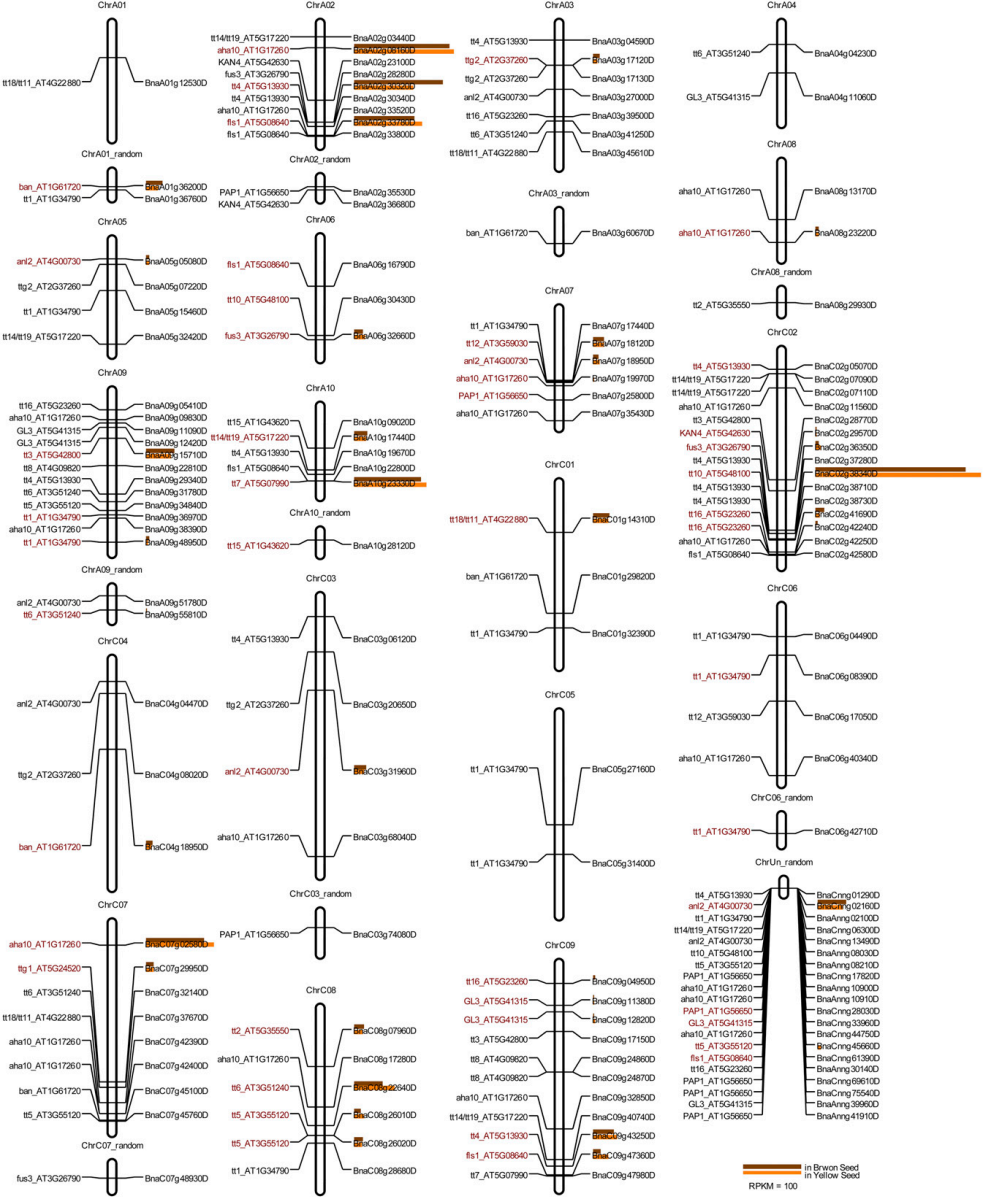
Transcription and *in silico* mapping of all the Arabidopsis *TT* genes orthologs in *B. napus*. Genes in red highlight the genes being transcribed in seed. Length of the brown and copper bar to the right of chromosome indicates transcription abundance in brown and yellow seeds respectively.

Current knowledge of seed coat pigment metabolism has been acquired largely from the model plant Arabidopsis. Based on *transparent testa* Arabidopsis mutants, about 24 genes have been elucidated in the flavonoid biosynthetic pathway (Yu 2013). Based on the released genome of *B. napus*, we *in silico* mapped a total of 139 orthologous genes of Arabidopsis *transparent testa* (*TT*) genes to 19 chromosomes of *B. napus* (Figure 6). In total, 60 orthologs map to the A genome, and 59 map to the C genome. The other 20 remain unassigned (Table S6). The number of orthologs of each Arabidopsis *TT* gene varies from 2 to 20 in *B. napus*, with 1 to 4 transcribed in seeds, except *TT8*, which has triple orthologs in ChrC9, but of which none are transcribed (Figure 6). Of all the *TT* orthologs in *B. napus*, 49 genes (about one-third) have transcription detected in seeds 26 DAP by transcriptome *de novo* sequencing. One of the *tt10* orthologs, *BnaC02g38340D*, is the most abundantly transcribed *TT* gene in the seeds 26 DAP of *B. napus*, with a transcriptional abundance comparable to that of *BnTub2* (*BnaC03g56170D*). However, none of the genes has significant difference of transcription between the wild type brown and the yellow mutant (Figure 6). The results indicate that transcription of *TT* gene orthologs is independent of this embryonal control of seed coat color. A novel gene may be responsible.

### qRT-PCR verification of the differentially expressed genes

**Figure 7:**
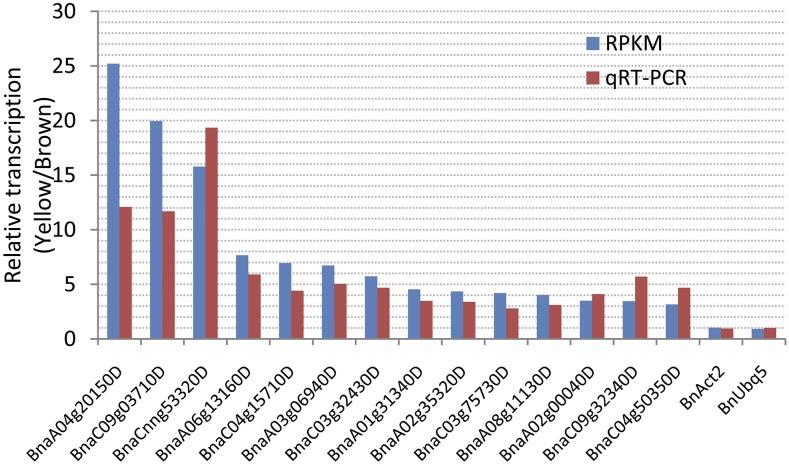
qRT-PCR verification of 23 upregulated genes detected by RNA-seq.

To confirm the difference in expression levels of the genes as revealed by RPKM analysis, 14 of the 50 upregulated genes were chosen for qRT-PCR analysis (Figure 7). RPKM analysis revealed that the differences in transcription levels varied from 2.31-fold to 25.21-fold. Consistently, qRT-PCR showed that the transcription difference varied from 2.79-fold to 19.62-fold. The correlation between qRT-PCR and RPKM analysis is high.

## Discussion

In this research, we found a yellow-allele-dominant locus exerting embryonal control of seed color in *B. napus*. The effect of this locus may be quite different to previously reported yellow seed related loci. In previous studies (Rahman *et al.* 2001, 2010; Li *et al.* 2012), all the alleles in yellow seeded plants were recessive to the brown seeded alleles at all of the seed color controlling loci in *B. napus*. Similar findings have also been confirmed in other *Brassica* species (Schwetka 1982; Padmaja *et al.* 2005). Even in *Arabidopsis*, all but one of the Arabidopsis *kan4* alleles are recessive in the 21 cloned testa-color-controlling loci (Routaboul *et al.* 2006; Yu 2013). Although some dominant QTL controlling yellow seed coat in *B. napus* have been mapped (Liu *et al.* 2012), all the loci exerted a maternal control effect on seed color. In a previous report (Heneen and Brismar 2001), the role of maternal and embryonal control of seed color in resynthesized *B. napus* was emphasized. However, the loci exerting embryonal control of yellow seed coat color were recessive. Further, the transcriptional abundance of current known *TT* homologous genes is independent of seed coat color. Thus, a novel gene may be responsible for the embryonal control of seed coat color in *B. napus*.

According to seed structure (Wan *et al.* 2002; Moise *et al.* 2005), seed coat develops from the integument that surrounds the ovule. The genome of these cells is completely derived from the maternal plant. The seed coat pigment is synthesized in the cells of the outer integument layer (Figure 2B). Therefore it is reasonable to conclude that the maternal plant genotype controls seed coat color generally. This is consistent with previous reports. However, how the embryo controls seed coat color is still unknown. In order for the embryo to control seed coat color, there must be communication between the cells of the integument and the embryo. Here, we report that disappearance of pigment in the seed coat is correlated with the accumulation of Ala and Phe in the embryo. Thus, we deduce that the communication likely involves Ala- and Phe-dependent chemicals.

**Figure 8:**
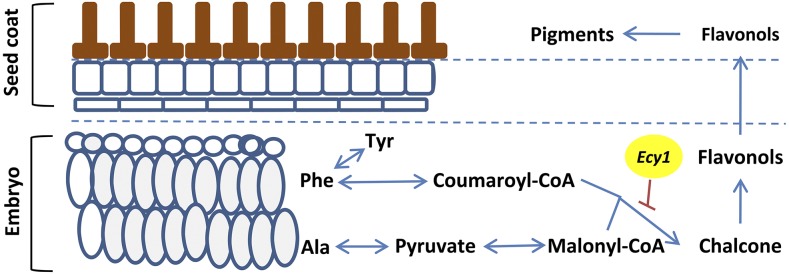
The model proposed for *Ecy1* controlling seed coat color in *B. napus*.

The relationship of Phe with pigment synthesis has been well documented (Winkel-Shirley 2001; Routaboul *et al.* 2006; Albert *et al.* 2014). For pigment synthesis, Phe must be metabolized into coumaroyl-CoA. One molecule of coumaroyl-CoA, and two molecules of malonyl-CoA, are combined for chalcone synthesis, which is the initial precursor of variant pigments (Winkel-Shirley 2001). According to the glycolysis pathway, pyruvate is a common substrate for Ala and coumaroyl-CoA synthesis. So the connection between Ala and coumaroyl-CoA is established. Interestingly, the ratio of Ala:Phe, which showed higher accumulation in yellow seed embryos than in brown seed embryos, is about 2:1—the formula for chalcone synthesis. The concentration of Phe and Ala in yellow seed embryos is 801.2 and 1537.7 µmol/g, respectively, but is 486.1 and 821.8 µmol/g in the brown seed embryo. Thus the extra amount of Phe and Ala in the yellow seed embryo is 315.1 and 715.9 µmol/g, respectively. Taking together the excessive amount of Tyr, which is exclusively metabolized from Phe, 40.1 µmol/g more in yellow embryo than in brown embryo, the overaccumulated Ala (715.9 µmol/g) is almost exactly twice the amount of Phe (315.1 µmol/g) plus Tyr (40.1 µmol/g) in the yellow embryo. So we believe that the pigment in the seed coat is synthesized from Phe and Ala in the embryo. Possible substrates transported from the embryo to the seed coat may be chalcone-derived flavonols. In Arabidopsis, some diglycosylated flavonols, such as quercetin-3-o-glucoside, and kaempferol-3,7-di-o- rhamnoside, were specifically identified in the embryo rather than in the seed coat (Routaboul *et al.* 2006). Based on our results, a novel model on the embryonal control of seed coat color in *B. napus* is proposed in Figure 8, and we assign the name *Ecy1* to the embryonal gene controlling seed coat color. *Ecy1* negatively regulates the synthesis of some chalcone derived flavonols, which are transported from embryo to seed coat for pigment synthesis, from Phe and Ala.

## 

## Supplementary Material

Supporting Information
