# Supplementary material for: Embryonal Control of Yellow Seed Coat Locus ECY1 Is Related to Alanine and Phenylalanine Metabolism in the Seed Embryo of Brassica napus
Source: G3 (Bethesda). 2016 Feb 18;6(4):1073–81. doi: 10.1534/g3.116.027110 (PMC4825642; doi:10.1534/g3.116.027110)
Supplement: Supporting Information [file supp_6_4_1073__index.html]

Embryonal Control of Yellow Seed Coat Locus ECY1 Is Related to Alanine and Phenylalanine Metabolism in the Seed Embryo of Brassica napus — Supporting Information 

# Embryonal Control of Yellow Seed Coat Locus *ECY1* Is Related to Alanine and Phenylalanine Metabolism in the Seed Embryo of *Brassica napus*

## Supporting Materials for Wang *et al.*, 2016

**Files in this Data Supplement:**

- Figure S1 - GO classification of unigenes in seeds of *B. napus*. (.pdf, 111 KB)
- Figure S2 - COG function classification of transcriptome of genes in seeds of *B. napus*. (.pdf, 183 KB)
- Figure S3 - KEGG classification of unigenes in seeds of *B. napus*. (.pdf, 193 KB)
- Table S1 - Sequence data generated by llumina HiSeqTM 2000. (.pdf, 49 KB)
- Table S2 - Genes assembled by Trinity. (.pdf, 40 KB)
- Table S3 - Annotation of assembled genes. (.pdf, 46 KB)
- Table S4 - Gene specific primers used for qPCR verification. (.xlsx, 21 KB)
- Table S5 - Free amino acids content in Cy2B and Cy2Y testa and embryo. (.xlsx, 15 KB)
- Table S6 - All the Arabidopsis transparent testa genes and the orthologs in *B. napus*. (.xlsx, 14 KB)
