# Supplementary figures and images for: Embryonal Control of Yellow Seed Coat Locus ECY1 Is Related to Alanine and Phenylalanine Metabolism in the Seed Embryo of Brassica napus
Source: G3 (Bethesda). 2016 Feb 18;6(4):1073–81. doi: 10.1534/g3.116.027110 (PMC4825642; doi:10.1534/g3.116.027110)

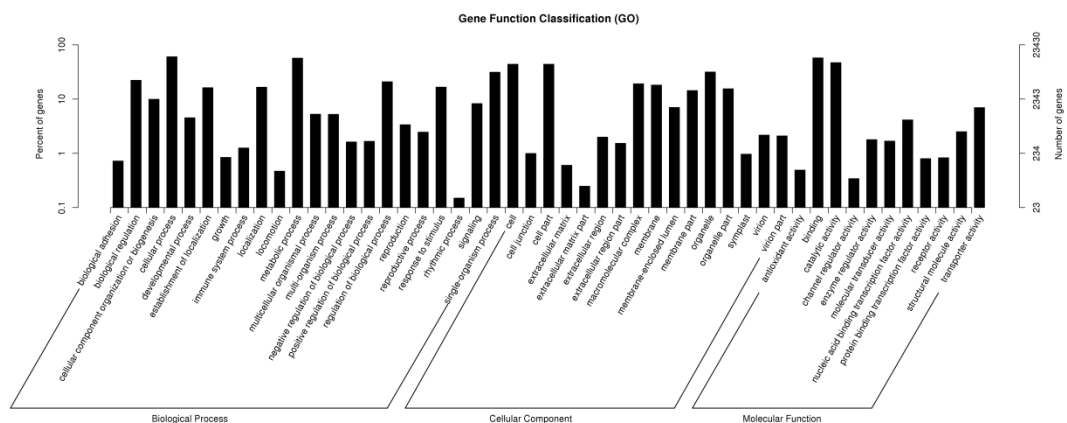

Figure S1: GO classification of unigenes in seeds of *B. napus*.

Supplement: Supporting Information [file supp_g3.116.027110_FigureS1.pdf]

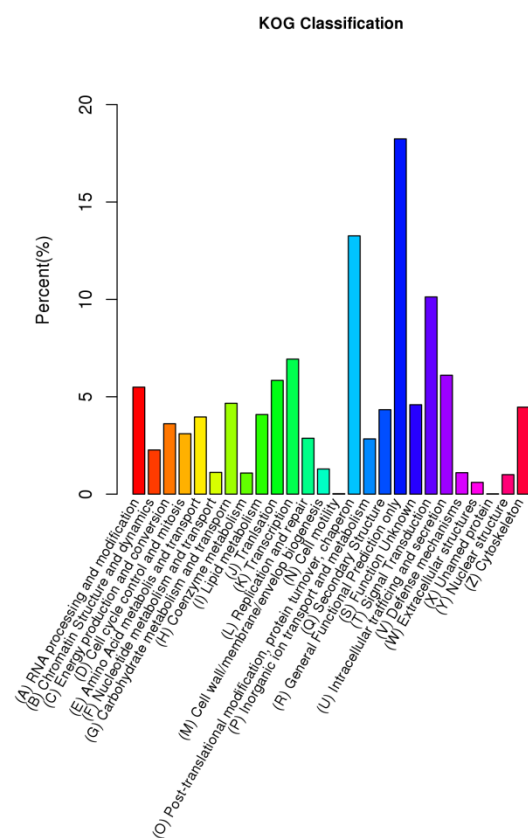

Figure S2: COG function classification of transcriptome of genes in seeds of *B. napus*.

Supplement: Supporting Information [file supp_g3.116.027110_FigureS2.pdf]

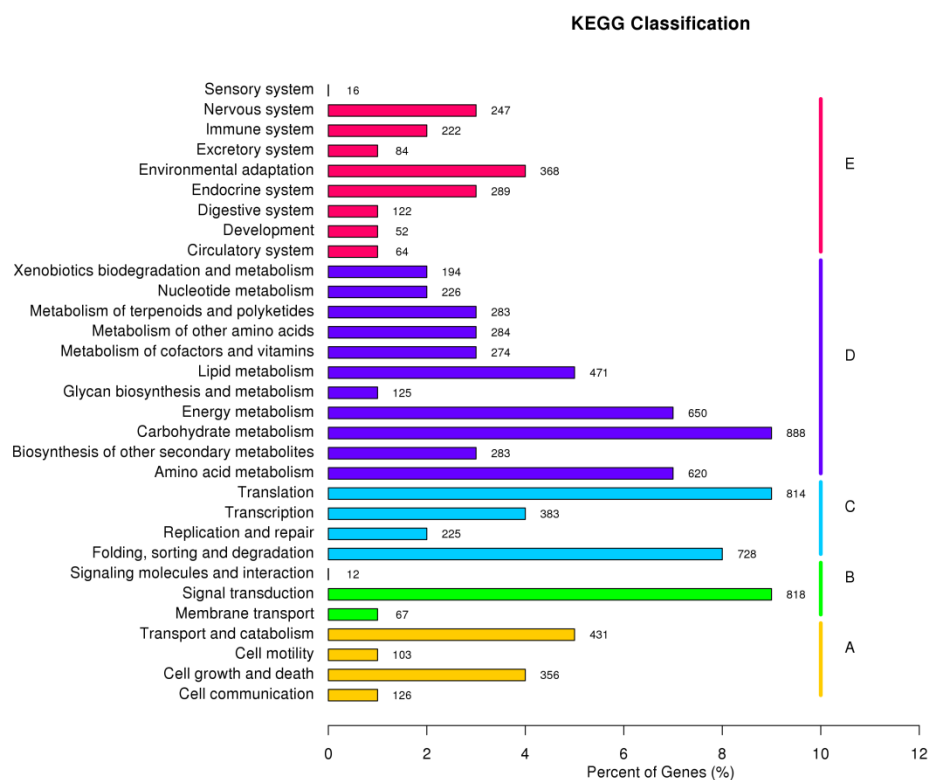

Figure S3: KEGG classification of unigenes in seeds of *B. napus*.

Supplement: Supporting Information [file supp_g3.116.027110_FigureS3.pdf]
