# Supplementary material for: Embryonal Control of Yellow Seed Coat Locus ECY1 Is Related to Alanine and Phenylalanine Metabolism in the Seed Embryo of Brassica napus
Source: G3 (Bethesda). 2016 Feb 18;6(4):1073–81. doi: 10.1534/g3.116.027110 (PMC4825642; doi:10.1534/g3.116.027110)
Supplement: Supporting Information [file supp_g3.116.027110_TableS1.pdf]

Table S1: Sequence data generated by llumina HiSeq^{TM} 2000

Sample	Raw Reads	Clean reads	Clean bases	Error(%)	Q20(%)	Q30(%)	GC(%)
L07_26d_1	31609633	31060834	3.11G	0.03	98.41	94.12	46.07
L07_26d_2	31609633	31060834	3.11G	0.03	97.76	93.03	46.02
L08_26d_1	31546496	30823125	3.08G	0.03	97.87	92.55	46.20
L08_26d_2	31546496	30823125	3.08G	0.03	97.37	91.88	46.16
