# Supplementary material for: Embryonal Control of Yellow Seed Coat Locus ECY1 Is Related to Alanine and Phenylalanine Metabolism in the Seed Embryo of Brassica napus
Source: G3 (Bethesda). 2016 Feb 18;6(4):1073–81. doi: 10.1534/g3.116.027110 (PMC4825642; doi:10.1534/g3.116.027110)
Supplement: Supporting Information [file supp_g3.116.027110_TableS2.pdf]

Table S2: Genes assembled by Trinity

	Min Length	Mean Length	Median Length	Max Length	N50	N90	Total Nucleotides
Transcripts	201	1155	898	16406	1699	558	198923288
Unigenes	201	784	404	16406	1414	292	43631916
