# Supplementary material for: Embryonal Control of Yellow Seed Coat Locus ECY1 Is Related to Alanine and Phenylalanine Metabolism in the Seed Embryo of Brassica napus
Source: G3 (Bethesda). 2016 Feb 18;6(4):1073–81. doi: 10.1534/g3.116.027110 (PMC4825642; doi:10.1534/g3.116.027110)
Supplement: Supporting Information [file supp_g3.116.027110_TableS3.pdf]

Table S3: Annotation of assembled genes

	Number of Unigenes	Percentage (%)
Annotated in NR	33736	60.63
Annotated in NT	31807	57.16
Annotated in KO	9490	17.05
Annotated in SwissProt	23309	41.89
Annotated in PFAM	20441	36.73
Annotated in GO	23430	42.11
Annotated in KOG	12088	21.72
Annotated in all Databases	4686	8.42
Annotated in at least one Database	41490	74.57
Total Unigenes	55637	100
